# Effectiveness of Interactive Audiovisual Training on Breastfeeding Knowledge Among Primary Healthcare Workers in Eastern India: A Quasi-experimental Study

**DOI:** 10.7759/cureus.79335

**Published:** 2025-02-19

**Authors:** G. Jahnavi, Bijit Biswas, Anuradha Gautam, Shikha Sahay, Saroj Kumar Tripathi, Jyoti Kiran

**Affiliations:** 1 Community and Family Medicine, All India Institute of Medical Sciences, Deoghar, IND; 2 Community Medicine, IQ City Medical College and Hospital, Durgapur, IND; 3 Obstetrics and Gynecology, All India Institute of Medical Sciences, Deoghar, IND; 4 Pediatrics, All India Institute of Medical Sciences, Deoghar, IND

**Keywords:** allied health personnel, awareness, breast feeding, early intervention, educational, india, primary health care

## Abstract

Background

Accredited Social Health Activists (ASHAs) are vital in promoting breastfeeding in rural areas. However, their understanding and misconceptions regarding this vital lifesaving intervention remain largely unexplored. The study sought to measure the effectiveness of a one-day interactive, audiovisual training on breastfeeding knowledge among ASHAs of a rural block of Deoghar, Jharkhand, India.

Methods

Utilizing a quasi-experimental design featuring a single intervention group, this study engaged 46 ASHAs from a rural block in Deoghar, Jharkhand, India, coinciding with World Breastfeeding Week in August 2023. The evaluation encompassed a broad range of sociodemographic variables along with an in-depth exploration of breastfeeding knowledge, spanning techniques, benefits, and common misconceptions.

Results

The study participants, with an average age of 39.3 years and a mean service duration of 15.6 years, included a majority (71.7%) who had received prior breastfeeding training. Analysis of pre- and post-intervention data revealed significant knowledge improvements, with an effect size (Cohen’s d) of 2.0, ranging between 1.5 and 2.5. Notably, participants with higher educational levels (correlation coefficient, r = 0.360, p = 0.014) and those with previous training (r = 0.535, p < 0.001) experienced substantial gains. In contrast, the improvements were less marked (r = -0.317, p = 0.032) among ASHAs from Scheduled Castes or Scheduled Tribes.

Conclusions

The effectiveness of interactive, audiovisual training highlights the importance of regular, context-specific education for ASHAs, enabling them to effectively support and advocate for breastfeeding in their communities.

## Introduction

Breastfeeding plays a pivotal role in the growth, development, and health of infants, making it a fundamental public health strategy to enhance infant survival rates globally [1,2]. Despite its acknowledged importance, there is a concerning trend in Jharkhand, India, as revealed by the National Family Health Survey (NFHS). Data shows a significant decline in immediate post-birth breastfeeding initiation rates, decreasing from 33.1% to 21.5%, in stark contrast to the rise in exclusive breastfeeding rates during the first six months from 64.8% to 76.1% [3,4]. Furthermore, the rates of early initiation and exclusive breastfeeding in Jharkhand (NFHS-4: 41.6% and 54.9%; NFHS-5: 41.8% and 63.7%, respectively) are substantially lower than India’s national averages [5,6]. These findings highlight the urgent need for targeted interventions to address these disparities and improve breastfeeding practices in Jharkhand.

Community health workers, often referred to as Accredited Social Health Activists (ASHAs) in certain regions, are essential in advancing breastfeeding, especially in rural and underserved communities [7,8]. They are crucial in informing mothers about the World Health Organization’s recommendation of exclusive breastfeeding for the first six months to promote infant survival and development [9]. Through their guidance and support, these health workers assist mothers in navigating breastfeeding challenges, merging traditional practices with contemporary health guidelines. Their contributions are key to enhancing maternal and child health outcomes. Research indicates variability in the knowledge levels of such community health workers concerning different aspects of breastfeeding, including initiation, importance, continuation, and management of potential issues [10-14].

Given the critical decline in immediate post-birth breastfeeding rates in Jharkhand, India, despite an increase in exclusive breastfeeding and the crucial role of ASHAs in enhancing breastfeeding practices, there was a clear necessity for interventional studies aimed at improving ASHAs’ knowledge. Previous research in India has demonstrated the positive impact of educational interventions on ASHAs’ understanding of home-based newborn care (HBNC) and infant and young child feeding (IYCF) [15,16]. However, a notable gap exists in the literature regarding such interventions in Jharkhand or Eastern India. To address this, the present study was conceived to coincide with World Breastfeeding Week, 2023. Its goal was to measure the effectiveness of a one-day interactive, audiovisual training on breastfeeding knowledge among ASHAs of a rural block of Deoghar, Jharkhand, India, thereby filling a critical gap in the research and supporting the improvement of breastfeeding rates and practices in the region.

## Materials and methods

Study design and setting

This quasi-experimental pre-post study was conducted in the Devipur block of Deoghar, Jharkhand, India, during World Breastfeeding Week 2023. The study employed a single experimental group without a control group to assess the effectiveness of a one-day interactive audiovisual training session on breastfeeding knowledge among ASHAs.

Sample size, sampling design, and participant recruitment

A total of 46 ASHAs were recruited for the study, surpassing the initial sample size requirement. This initial calculation aimed to measure the effectiveness that a one-day interactive audiovisual training session could have on the participants’ knowledge about breastfeeding. The parameters set for this assessment included an anticipated effect size of at least 0.5, a precision of within 5%, and a statistical power of 80%. These parameters led to the determination that a minimum of 34 participants would be needed, as calculated by Statulator, an online tool designed for sample size estimation [17]. To ensure an unbiased selection, the medical officer of the block hospital was requested to arrange for the participation of at least 50 ASHAs from the 301 ASHAs working in the block. The selection process was independent of the researchers to ensure unbiased participant recruitment.

Data collection instrument

Data were collected using a structured questionnaire that captured two primary areas: participants’ background characteristics and their breastfeeding knowledge. Background characteristics included age, duration of service, marital status, caste, religion, educational level, monthly family income, and prior training in breastfeeding. The breastfeeding knowledge section comprised 28 items meticulously developed from an extensive literature review [1,18-20]. Domains covered included breastfeeding initiation and continuation, correct positioning and attachment, managing complications, understanding the benefits of breastfeeding, and addressing common misconceptions. Each correct response was awarded one point, with reverse scoring applied to specific items (K-20 to K-25 and K-28) to enhance reliability. The questionnaire demonstrated good internal consistency, with Cronbach’s alpha values ranging from 0.739 to 0.754.

Intervention

The training session, held in the block hospital’s conference hall, spanned three hours and followed a structured approach. It began with a 15-minute pre-test to assess participants’ baseline knowledge, followed by an interactive 150-minute session incorporating PowerPoint presentations and educational videos, and concluded with a 15-minute post-test to evaluate learning outcomes. The facilitator actively engaged participants, addressing their queries and clarifying doubts regarding breastfeeding. Experts from the Departments of Community and Family Medicine and Obstetrics and Gynecology led the session, emphasizing the nutritional and immunological benefits of breastfeeding while covering practical aspects such as early initiation, correct positioning, and safe storage of breast milk. To enhance learning, two educational open-source videos were incorporated into the session. The first video focused on the benefits of breastfeeding for both mother and child, highlighted practical challenges faced by breastfeeding mothers, and underscored the critical role of healthcare workers in providing support (Appendix A). The second video provided guidance on expressing and storing breast milk, particularly for working mothers (Appendix B). These audiovisual resources supplemented the training, making it more engaging and informative.

Ethical considerations

The study was approved by the Institutional Ethics Committee (IEC) at the All India Institute of Medical Sciences (AIIMS), Deoghar, under reference number 2023-219-IND-03. Data collection was integrated into the World Breastfeeding Week 2023 event. Informed written consent was obtained from all participants, and stringent measures were taken to ensure confidentiality and anonymity throughout the study.

Statistical analysis

Data collection was digitized using Google Forms (Google LLC, Mountain View, CA, USA) to minimize errors, and the compiled data was exported to linked Excel sheets (Microsoft Corporation, Redmond, WA, USA) for analysis using JAMOVI (version 2.3.28) [21]. McNemar’s chi-square test was employed to evaluate changes in individual knowledge items before and after the intervention. The normality of pre- and post-intervention knowledge scores was evaluated using a Q-Q plot and the Shapiro-Wilk test. Overall changes in knowledge scores were analyzed using the paired t-test, with effect size estimated through Cohen’s d and reported with a 95% CI. Additionally, Spearman’s rho correlation analysis was conducted to identify factors significantly influencing knowledge gains. A p-value of less than 0.05 was considered statistically significant.

## Results

Most of the study’s participants were aged between 30 and 39 years, with an average age of 39.3 years and a SD of 7.3 years. A significant majority (73.9%) had been working as ASHA workers for 15 to 19 years, with an average tenure of 15.6 years (SD = 5.4 years). In terms of educational background, 47.8% completed middle school, while 8.7% held graduate degrees. More than two-thirds (71.7%) reported having received training related to breastfeeding. Before the intervention, knowledge about various aspects of breastfeeding varied widely, with 6.5% to 65.2% for positively scored attributes and 30.4% to 71.7% for negatively scored items. After the intervention, knowledge levels for positively scored attributes ranged from 30.4% to 84.8% and for negatively scored items, from 2.2% to 32.6%. Significant improvements were observed in all assessed areas except for questions related to continued breastfeeding and breastfeeding in the context of HIV positive mothers after the educational intervention (Table 1, Table 2).

**Table 1:** Background characteristics of the study participants (n = 46) All the study participants were females. ASHA: Accredited Social Health Activist; OBC: Other Backward Caste; SC: Scheduled Caste; ST: Scheduled Tribe

Variable	n (%)
Age (in completed years)	
<30	4 (8.7)
30-39	19 (41.3)
40-49	17 (37.0)
≥50	6 (13.0)
Duration since working as ASHA (in years)	
<10	7 (15.2)
10-14	2 (4.3)
15-19	34 (73.9)
≥20	3 (6.5)
Marital status	
Currently married	42 (91.3)
Widow	3 (6.5)
Divorced	1 (2.2)
Religion	
Hindu	43 (93.5)
Muslim	3 (6.5)
Caste	
General	15 (32.6)
OBC	21 (45.7)
SC	5 (10.9)
ST	5 (10.9)
Educational level	
Middle	22 (47.8)
Secondary	2 (4.3)
Higher secondary	18 (39.1)
Graduate	4 (8.7)
Monthly family income (in USD)	
<60.3	12 (26.1)
60.3-120.5	29 (63.0)
≥120.6	5 (10.9)

**Table 2:** Item-wise effect of one-day interactive educational intervention on breastfeeding-related knowledge among ASHAs (n = 46) ^*^ McNemar’s chi-square test ASHA: Accredited Social Health Activist

Item	Variable	Correct response	Before (%)	After (%)	p-Value^*^
K1	Breastfeeding should be initiated within an hour after birth.	True	65.2	78.3	0.034
K2	Exclusive breastfeeding should be done up to the first six months after birth.	True	56.5	84.8	<0.001
K3	Continued breastfeeding should be done for two years.	True	56.5	71.7	0.09
K4	Could name all the steps of breastfeeding	Yes	13	58.7	<0.001
K5	Could name all the steps of positioning during breastfeeding	Yes	23.9	65.2	<0.001
K6	Could name all the steps of nipple attachment	Yes	26.1	63	<0.001
K7	Breastmilk is adequate for the nutritional requirements of a child until six months.	True	39.1	73.9	<0.001
K8	Expressed breastmilk can be safely stored for six to eight hours at room temperature.	True	13	58.7	<0.001
K9	Breastfeeding promotes the brain development of the child.	True	8.7	47.8	<0.001
K10	Breastfeeding boosts the immunity of the child.	True	19.6	60.9	<0.001
K11	Breastfed child has a lower risk of asthma in future life.	True	8.7	39.1	<0.001
K12	Breastfed child has a lower risk of obesity in future life.	True	17.4	41.3	<0.001
K13	Breastfed child has a lower risk of type 2 diabetes in future life.	True	6.5	30.4	<0.001
K14	Breastfeeding helps in uterine involution of the mother.	True	19.6	45.7	<0.001
K15	Breastfeeding reduces the risk of breast cancer in mother.	True	10.9	34.8	<0.001
K16	Breastfeeding reduces the risk of ovarian cancer in mother.	True	8.7	34.8	<0.001
K17	Breastfeeding facilitates postpartum weight loss.	True	19.6	39.1	0.003
K18	A sore nipple is one of the common problems associated with breastfeeding.	True	19.6	47.8	<0.001
K19	Breast engorgement is one of the common problems associated with breastfeeding.	True	10.9	34.8	<0.001
K20	Insufficient breast milk production is one of the common problems associated with breastfeeding.	False	54.3	10.9	<0.001
K21	Colostrum is not beneficial for the child; therefore, it should not be fed.	False	69.6	2.2	<0.001
K22	Introducing animal milk (such as cow’s milk) alongside breast milk during the first six months (mixed feeding) can promote a child’s growth.	False	69.6	23.9	0.001
K23	If a mother is malnourished, her milk will not provide enough nutrients for the baby.	False	30.4	10.9	0.039
K24	The mother should stop breastfeeding if the child gets diarrhea.	False	47.8	13	0.002
K25	A child should be given water along with breastmilk during the first six months (especially during summer) to avoid dehydration.	False	71.7	8.7	<0.001
K26	Lactational amenorrhea is an effective way of contraception.	True	26.1	52.2	<0.001
K27	One breast should be emptied before initiating feeding from another breast.	True	17.4	43.5	<0.001
K28	HIV-positive mother should not breastfeed her child.	False	39.1	32.6	0.083

**Figure 1:**
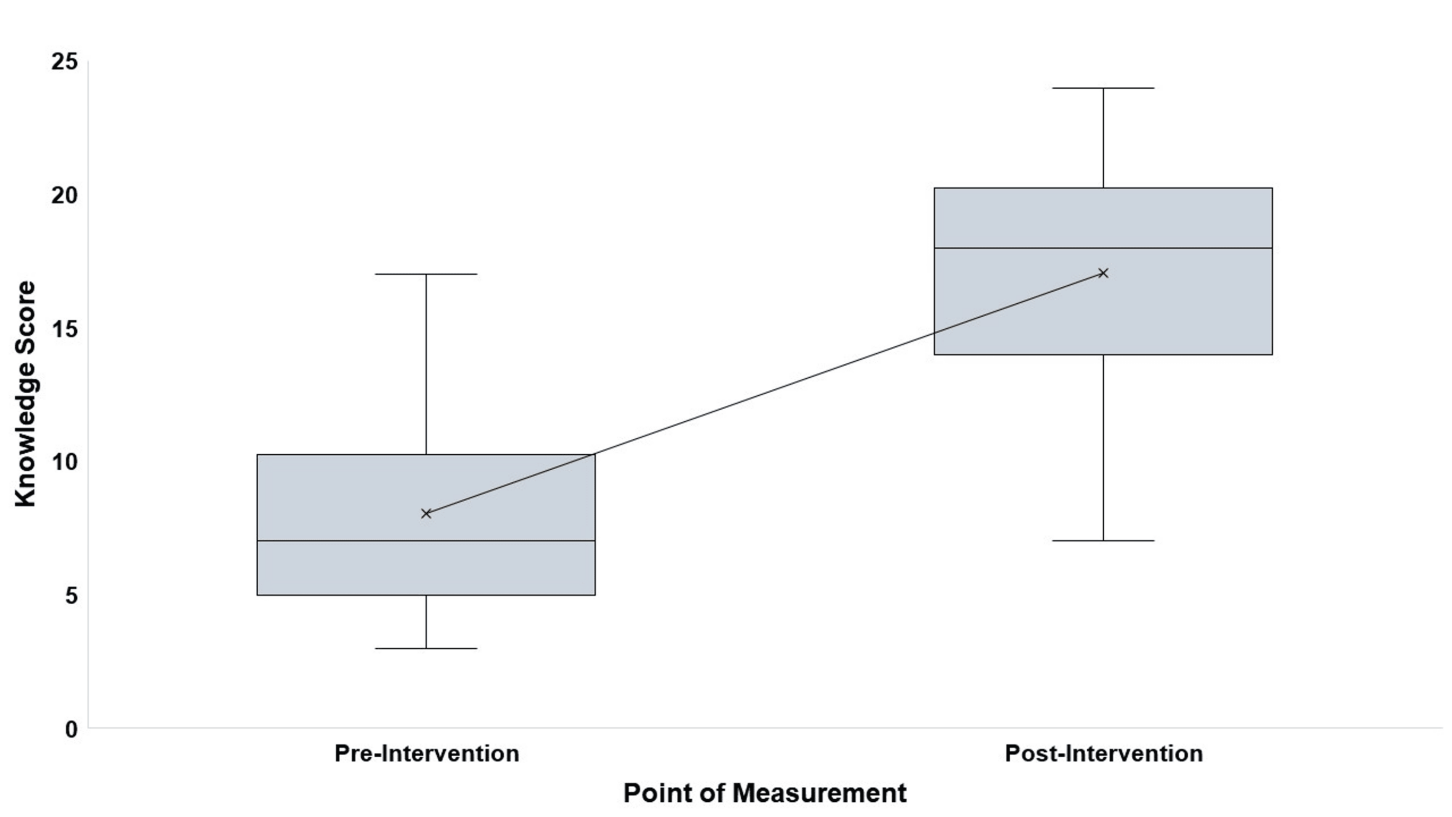
Box whisker plot showing the distribution of pre- and post-educational intervention knowledge score of ASHAs (n = 46) ASHA: Accredited Social Health Activist

Although the post-test knowledge score (p = 0.051) and the difference between post- and pre-test knowledge scores (p = 0.097) followed a normal distribution, the pre-test knowledge score did not (p = 0.008). The mean knowledge score on breastfeeding increased substantially from 8.0 (SD = 3.7) before the intervention to 17.0 (SD = 4.6) after the intervention, reflecting a mean improvement of 9.0 (SD = 4.6) points. This significant gain corresponds to a large effect size of 2.0 (95% CI: 1.5 to 2.5), indicating a highly significant enhancement in knowledge (p < 0.001) (Table 3, Figure 1).

**Table 3:** Overall effect of one-day interactive educational intervention on breastfeeding-related knowledge among ASHAs (n = 46) ^*^ Cohen’s d ^#^ Paired t-test ASHA: Accredited Social Health Activist

Variable	Mean ± SD	Mean difference ± SD	Effect size (95% CI)^*^	p-Value^#^
Pre-test knowledge score	8.0 ± 3.7	9.0 ± 4.6	2.0 (1.5-2.5)	<0.001
Post-test knowledge score	17.0 ± 4.6			

Participants with a higher level of education (correlation coefficient, r = 0.360, p = 0.014) and those who had previously received training on breastfeeding (r = 0.535, p < 0.001) were more likely to exhibit significant gains in breastfeeding-related knowledge following the training. In contrast, individuals from Scheduled Castes (SCs) or Scheduled Tribes (STs) (r=-0.317, p=0.032) experienced a lesser increase in knowledge on the subject compared to their counterparts (Table 4).

**Table 4:** Correlation between background characteristics and effect of educational intervention among the study participants (n = 46) ^*^ Spearman’s rho correlation test ASHA: Accredited Social Health Activist, SC: Scheduled Caste, ST: Scheduled Tribe

Variable	Correlation coefficient	p-Value^*^
Age in completed years (increasing)	-0.148	0.327
Duration since working as ASHA (increasing)	0.08	0.597
Marital status (married)	-0.064	0.672
Religion (Hindu)	0.057	0.709
Caste (SC/ST)	-0.317	0.032
Educational level (increasing)	0.36	0.014
Monthly family income (increasing)	0.088	0.559
Received prior training on breastfeeding (yes)	0.535	<0.001

## Discussion

This was a facility-based, quasi-experimental study conducted among selected ASHAs in a rural block of Deoghar, Jharkhand, India. It employed an audiovisual, interactive one-day intervention. Through our intervention, we were able to achieve a large effect size. Educational level, caste, and prior training status emerged as significant attributes affecting the effectiveness of the intervention.

Prior to the intervention, there was a wide range of knowledge about various aspects of breastfeeding among participants. For positively scored attributes, the understanding varied from as low as 6.5% for the belief that breastfeeding lowers the risk of type 2 diabetes to as high as 65.2% for knowledge of the appropriate time to initiate breastfeeding. For negatively scored items, awareness ranged from 30.4%, concerning the misconception that milk from malnourished mothers is of poor quality, to 71.7%, relating to the unnecessary provision of extra water during exclusive breastfeeding. These figures are notably lower when compared to findings from a cross-sectional study by Saxena et and Kumari [22] in Doriwala block, Dehradun, Uttarakhand, where knowledge spanned from 44.6% regarding the age of weaning to 98.0% on the definition of exclusive breastfeeding. Similarly, a study by Pareek et al. [23] among ASHAs of the Panvel block in Raigad district, Maharashtra, reported knowledge levels ranging from 20.0% on the disadvantages of formula feeding to 100.0% on the initiation of breastfeeding after birth. Another research by Rohith and Angadi [13] among ASHAs from three Taluks in Vijayapur district, Karnataka, found knowledge levels between 80.3% for initiation of breastfeeding and 94.0% for the definition of exclusive breastfeeding. Furthermore, a study by Saxena et al. [11] in Bhojipura block, District Bareilly, Uttar Pradesh, reported a range from 23.4% on prelacteal feed to 95.3% on the initiation of breastfeeding. When our results are compared with the broader population, a study by Pandey et al. [12], which compared knowledge on breastfeeding between two generations of Indian women (pregnant women and their mothers-in-law) in Karnataka, found it varied from 16.4% on the components of breastmilk to 97.7% on identifying the best food for a newborn. This comparison underscores the substantial variability and gaps in breastfeeding knowledge, highlighting the critical need for targeted educational interventions.

Following the intervention, we observed an improvement in knowledge levels for positively scored attributes, which ranged from 30.4% for the understanding that breastfeeding reduces the risk of type 2 diabetes in a breastfed child’s future life to 84.8% for the awareness that exclusive breastfeeding is recommended for the first six months after birth. For negatively scored items, misconceptions decreased to range from 2.2% for the belief that colostrum is not beneficial for the child to 32.6% for the misconception that HIV-positive mothers should not breastfeed. Our audiovisual interactive intervention successfully achieved a significant effect size, mirroring the outcomes seen in similar studies. For instance, Gauba and Singh [15] reported that after an intervention (Structured Training Program on Newborn Care Based on ASHA Module 7) among ASHAs of a selected community in Delhi, there was a notable increase in the mean post-test knowledge score regarding breastfeeding to 7.8 from a baseline of 3.3. Similarly, Ojha et al. [24], in their study within the Panvel Municipal Corporation in Maharashtra, documented significant improvements in knowledge regarding IYCF following a one-day educational intervention. Furthermore, Devi et al. [10] conducted research among ASHAs in the Doriwala district of Dehradun, Uttarakhand, employing the Multiple Observation Method (a single-group time-series design). This study revealed that repeated educational interventions - administered four times with a 30-day interval between each session - significantly improved their knowledge of HBNC, including aspects of IYCF. A parallel study focusing on HBNC conducted by Chaudhary et al. [16] in Mandi, Himachal Pradesh, yielded comparable results, further corroborating the positive impact of regular and structured educational interventions on enhancing the proficiency of health workers in crucial areas of newborn and infant care.

In our study, the age of participants and their tenure as ASHAs showed an inverse relationship with the impact of the intervention. Specifically, age was negatively correlated, while work experience demonstrated a positive correlation with the intervention’s effectiveness. However, it is important to note that these correlations were not statistically significant. This finding contrasts with the research by Gauba and Singh [15], which identified both age and years of experience as significant factors influencing the success of educational interventions on HBNC among ASHAs. Educational level emerged as a significant factor affecting the intervention’s effectiveness, aligning with the observations made by both Gauba and Singh [15] and Chaudhary et al. [16] Moreover, the intervention’s impact was less pronounced among participants belonging to SC/ST, possibly due to a lower average educational level within these groups. This underscores the critical importance of considering educational background in the design and delivery of interventions. Participants who had received prior training on breastfeeding benefited more from our study’s intervention, highlighting the value of continuous and targeted re-education for ASHAs on breastfeeding practices. This finding suggests the need for regular sensitization efforts to ensure ASHAs are well informed and equipped to support breastfeeding effectively. Although we observed a positive correlation between family income and the intervention’s effectiveness, this correlation was not statistically significant. This diverges from the findings of Chaudhary et al. [16], which reported family income as a significant determinant of knowledge gain on HBNC through educational interventions. These discrepancies emphasize the complex interplay of socioeconomic and educational factors in determining the efficacy of health education programs, suggesting that tailored approaches may be required to optimize outcomes across diverse groups.

Our study provides insights into the potential of educational interventions to enhance ASHAs’ knowledge of breastfeeding practices. However, it faces limitations due to its small sample size and restriction to a single rural block, primarily for feasibility reasons. This limitation affects the generalizability of our findings across different demographics and urban settings. Additionally, the reliance on self-reported measures for assessing knowledge introduces bias, such as the tendency of participants to overestimate their understanding due to social desirability. The lack of long-term follow-up further complicates our understanding of the sustainability of the interventions’ effects on knowledge retention and practical application. The necessity for additional research is evident, especially in the form of longitudinal studies that evaluate the impact of diverse educational interventions, differentiated by their frequency and methodologies, on breastfeeding knowledge and practices. It is crucial that these studies also investigate the obstacles to implementation, providing a richer understanding of how to refine and optimize these interventions for greater effectiveness.

## Conclusions

Our study reveals that interactive, audiovisual training significantly improved breastfeeding knowledge among ASHAs, particularly those with higher educational levels and prior breastfeeding training. However, ASHAs from SCs and STs showed smaller gains, indicating a need for additional support and tailored interventions due to their disadvantaged backgrounds. These findings underscore the necessity for ongoing, context-specific educational programs that equip all ASHAs with the skills needed to effectively promote breastfeeding. Enhanced knowledge among ASHAs is crucial, as it might directly translate to improved breastfeeding practices, which are vital for reducing infant morbidity and mortality and improving overall maternal and child health in their communities.

## Appendices

Appendix A

 Appendix B

 Appendix C

**Table 5:** Knowledge regarding breastfeeding questionnaire

K1	Breastfeeding should be initiated within an hour after birth	True / False
K2	Exclusive breastfeeding should be done up to first 6 months after birth	True / False
K3	Continued breastfeeding should be done till 2 years	True / False
K4	Could you name the steps of breastfeeding:	
	Correct positioning of the baby	Yes / No
	Correct latching of the baby	Yes / No
	Sucking and milk let-down	Yes / No
	Unlatching the baby	Yes / No
	Burping	Yes / No
K5	Could name the steps of positioning during breastfeeding:	-
	Should be done in a comfortable position for the mother.	True / False
	Baby’s head and body should be straight.	True / False
	Baby’s face should face mother’s breast.	True / False
	Baby’s nose should be on the opposite side of the mother’s breast nipple.	True / False
	Baby should not be well supported from his/her bottom	True / False
K6	Could you name all the steps of nipple attachment:	-
	The baby’s chin should touch the breast.	True / False
	The baby’s mouth should be wide open.	True / False
	The baby’s lower lip should be turned inwards.	True / False
	One should be able to see more of areola above the Baby’s mouth than below	True / False
K7	Breastmilk is adequate for nutritional requirements of a child till six months	True / False
K8	Expressed breastmilk can be safely stored for 6-8 hours in room temperature	True / False
K9	Breastfeeding promotes brain development of the child	True / False
K10	Breastfeeding boosts immunity of the child:	True / False
K11	Breastfeed child has lower risk of Asthma in future life	True / False
K12	Breastfeed child has lower risk of Obesity in future life	True / False
K13	Breastfeed child has lower risk of Type 2 Diabetes in future life	True / False
K14	Breastfeeding helps in uterine involution of mother	True / False
K15	Breastfeeding reduces risk of breast cancer in mother	True / False
K16	Breastfeeding reduces risk of ovarian cancer in mother	True / False
K17	Breastfeeding facilitates postpartum weight loss	True / False
K18	Sore nipple is one of the common problems associated with breastfeeding	True / False
K19	Breast engorgement is one of the common problems associated with breastfeeding	True / False
K20	Insufficient breast milk production is one of the common problems associated with breastfeeding	True / False
K21	Colostrum is not beneficial for the child, therefore should not be fed	True / False
K22	Introducing animal milk (such as cow's milk) alongside breast milk during the first six months (mixed feeding) can promote a child's growth	True / False
K23	If a mother is malnourished, her milk will not provide enough nutrients for the baby	True / False
K24	Mother should stop breastfeeding if the child gets diarrhea	True / False
K25	A child should be given water along with breastmilk during the first six months (especially during summers) to avoid dehydration	True / False
K26	Lactational amenorrhea is an effective way of contraception	True / False
K27	One breast should be emptied before initiating feeding from another breast	True / False
K28	HIV positive mother should not breastfeed her child	True / False

## References

[1] (2024). The impact of breastfeeding on maternal and child health. https://www.unicef.org.uk/babyfriendly/news-and-research/baby-friendly-research/infant-health-research/infant-health-research-meta-analyses/the-impact-of-breastfeeding-on-maternal-and-child-health/.

[2] Horta BL (2019). Breastfeeding: investing in the future. Breastfeed Med.

[3] (2024). National Family Health Survey—4 (2015-2016): Jharkhand Fact Sheet. http://rchiips.org/nfhs/pdf/NFHS4/JH_FactSheet.pdf.

[4] (2024). National Family Health Survey—4 ( 2015-2016): India Fact Sheet. https://rchiips.org/nfhs/pdf/nfhs4/india.pdf.

[5] (2024). National Family Health Survey—5 ( 2019-2021): India Fact Sheet. https://rchiips.org/nfhs/NFHS-5_FCTS/India.pdf.

[6] (2024). National Family Health Survey—5 ( 2019-2021): Jharkhand Fact Sheet. https://rchiips.org/nfhs/nfhs-5_fcts/COMPENDIUM/Jharkhand.pdf.

[7] Imdad A, Yakoob MY, Bhutta ZA (2011). Effect of breastfeeding promotion interventions on breastfeeding rates, with special focus on developing countries. BMC Public Health.

[8] Namasivayam V, Dehury B, Prakash R (2021). Association of prenatal counselling and immediate postnatal support with early initiation of breastfeeding in Uttar Pradesh, India. Int Breastfeed J.

[9] (2024). Exclusive breastfeeding for optimal growth, development and health of infants. https://www.who.int/tools/elena/interventions/exclusive-breastfeeding.

[10] Devi RS, Pugazhendi S, Juyal R, Gaur A, Singh SB (2023). Evaluation of existing Home Based Newborn Care (HBNC) services and training for improving performance of Accredited Social Health Activists (ASHA) in rural India: a multiple observation study. Midwifery.

[11] Saxena S, Singh AK, Maheshwari S, Gupta SB. (2017). Appraisal of knowledge of ASHA regarding child health services provided under NHM in Bhojipura block, District Bareilly. Int J Community Med Public Health.

[12] Pandey D, Sardana P, Saxena A, Dogra L, Coondoo A, Kamath A (2015). Awareness and attitude towards breastfeeding among two generations of Indian women: a comparative study. PLoS ONE.

[13] Rohith M, Angadi MM (2020). Evaluation of knowledge and practice of ASHAs, regarding child health services in Vijyapaura District, Karnataka. J Family Med Prim Care.

[14] Bansal SC, Nimbalkar SM, Shah NA, Shrivastav RS, Phatak AG (2016). Evaluation of knowledge and skills of home based newborn care among Accredited Social Health Activists (ASHA). Indian Pediatr.

[15] Gauba A, Singh M (2021). A quasi-experimental study to assess the effectiveness of a structured training program on newborn care based on ASHA Module 7 - "Skills That Saves Lives" in terms of reported practice among ASHA workers in a selected community of Delhi. Indian J Community Med.

[16] Chaudhary S, Kumaran M, Regu M, Usha Usha, Lata C (2023). Effectiveness of educational program on knowledge and expressed practice regarding home based new born care among ASHA workers at District Mandi Himachal Pradesh. J ReAttach Ther Dev Divers.

[17] Singh Singh, N. D. and M. (n.d (2024). Sample size calculator for comparing paired differences. https://statulator.com/SampleSize/ss2PM.html.

[18] Horta BL, Victora CG (2013). Short-term effects of breastfeeding: a systematic review on the benefits of breastfeeding on diarrhoea and pneumonia mortality. https://iris.who.int/handle/10665/95585.

[19] Bernardo H, Cesar V (2013). Long-term effects of breastfeeding: a systematic review. https://iris.who.int/handle/10665/79198.

[20] Dieterich CM, Felice JP, O'Sullivan E, Rasmussen KM (2013). Breastfeeding and health outcomes for the mother-infant dyad. Pediatr Clin North Am.

[21] (2024). jamovi Desktop. https://www.jamovi.org/download.html.

[22] Saxena V, Kumari R (2014). Infant and young child feeding - knowledge and practices of asha workers of Doiwala Block, Dehradun District. Indian J Community Health.

[23] Pareek P, Waingankar P, Tawade A (2023). Knowledge and perception of Accredited Social Health Activists’ about breastfeeding and complementary feeding practices: a qualitative study using focus group discussion. Bharati Vidyapeeth Med J.

[24] Ojha S, Bandyopadhyay K, Patankar F, Rathod U (2022). Effectiveness of training programme on infant and young child feeding practices for Accredited Social Health Activist (ASHAs) and ANMs working in health department of Panvel Municipal Corporation in Maharashtra, India. Int J Med Res Health Sci.

